# Sulphites in the Treatment of Fevers

**Published:** 1868-07

**Authors:** A. C. Simonton

**Affiliations:** Mitchellville, Iowa


ARTICLE XXVI.

### SULPHITES IN THE TREATMENT OF FEVERS.

By A. C. SIMONTON, M.D,, Mitchellville, Iowa.

EDITOR CHICAGO MEDICAL EXAMINER,

*Dear Sir:*—As additional testimony in favor of the efficacy of sulphites in the treatment of *malarial diseases*, permit me to report the following cases through the columns of your journal:

CASE I, was that of myself. Having lived in one of the most excessively malarious districts on the Wabash River, in Indiana, for a number of years, and being rather susceptible to the influence of that poison, I found myself compelled to resort to the use of medicines every autumn, in order that I might enjoy anything like health. But for the last four or five years my system seemed to have become surcharged with the poison, and had taken on that condition which might properly be called a *malarious diathesis.* I was compelled, at least during all those seasons of the year when intermittents and remittents were rife, to resort to some remedy, *constantly*, as a prophylactic. I gave *arsenic* a thorough trial, and all those remedies vaunted as specifics, almost, in *chronic ague*, but to no avail; I could not rid it out of my system. I finally fell back to quinine, as the chief of its class, and continued taking it as a prophylactic, from one to three times a day, for at least half of the year. I went to Chicago, October 1st, 1867, to attend Chicago Medical College, and took with me nearly an ounce of quinine, of which I consumed the greater portion up to January, when, after hearing your lectures on the use of the sulphites in malarial diseases, I resolved to give them a thorough testing in my case. I therefore left off the quinine January 13th, 1868, and commenced taking the sulphite of soda in 10 gr. doses, four times a day, in solution.

I am happy to say that, in the space of one week, my appetite commenced improving, digestion was performed with more vigor, and that almost constant tendency to yawn and stretch, which are characteristic of this disease, had disappeared. In the space of about two weeks, I could feel no effects of the poison in my system; but, in order to make assurance "doubly sure," I continued the remedy for another fortnight, when I discontinued it, feeling almost like another person.

I took another short "course" of the sulphite in five or six weeks from the above-mentioned time, and have enjoyed perfect immunity from any of the symptoms of malarial poisoning up to this date, June 9th. Since I came to this locality, I have treated several cases of *chronic ague* with the sulphite of soda, successfully.

CASE II. D. D., farmer, who has been incapacitated for business for the past six months, owing to the frequent recurrence of intermittent fever, which, he says, has been broken up time after time, by the usual remedies, applied for treatment. Notwithstanding his assertion that he had taken an abundance of quinine, I ordered a sufficient quantity of that remedy to break the paroxysms, and then followed at once with the sulphite, in 10 gr. doses, four times a day. Had this continued for the space of three weeks, when the patient reported himself cured, and has since gone about his work with the vigor of a healthy man.

CASES III and IV of my note-book, are parallel with the one just reported; and there are others of different degrees of severity; but I will not occupy space in detailing them here.

Suffice it to say that, so far as my experience has gone, the sulphites are the remedies, *par excellence*, to rid the system of that poison, the nature and *modus operandi* of which is not yet fully established, which causes intermittent and remittent fevers. In all acute attacks I should deem it advisable, first, to check the paroxysms by the usual antiperiodics, after which, the remedy under consideration will complete the work.

I shall give this remedy a more thorough testing during the autumn months, and will probably report on some future occasion.

